# Musculoskeletal ultrasound findings of articular manifestations on juvenile primary sjogren’s syndrome

**DOI:** 10.1186/1546-0096-12-S1-P37

**Published:** 2014-09-17

**Authors:** Kosuke Shabana, Nami Okamoto, Keisuke Shindo, Takuji Murata, Hiroshi Tamai, Kenta Fujiwara

**Affiliations:** 1 Department of Pediatrics, Osaka Medical College, Takatsuki City, Japan; 2 Department of Orthopedic, Osaka Medical College, Takatsuki City, Japan

## Introduction

Articular manifestations (joint swelling, joint tenderness) are common extra-glandular manifestations of primary Sjogren’s syndrome (SjS). In past studies those have been reported that anti-cyclic citrullinated peptide antibody (ACPA) is associated with arthritis in adult SjS and that there is no clear method to distinguish arthritis of primary SjS from early rheumatoid arthritis, but there is no report on children. Musculoskeletal ultrasound (MSUS) can clearly evaluate arthritis or enthesitis which are difficult to assess in detail by only physical examination.

## Objectives

To investigate MSUS findings in juvenile primary SjS having articular manifestations with or without ACPA.

## Methods

Subject patients are 8 children who were diagnosed as juvenile primary SjS with articular manifestations between February 2013 and March 2014. We retrospectively evaluated sex, age of disease onset, disease duration, physical findings, blood examination data (rheumatoid factor and autoantibody) and MSUS findings from clinical records. All patients underwent salivary gland biopsy and satisfied 2012 American College of Rheumatology classification criteria for SjS. Joints (shoulder, sternoclavicular, elbow, wrist, metacarpophalangeal, proximal interphalangeal, hip, knee, ankle, metatarsophalangeal) and 5 entheseal sites of the lower limbs were scanned by a trained physician.

## Results

In 8 patients (7 females and 1 male), mean age of disease onset was 10.9 ± 3.3 years old. Mean disease duration was 0.9 ± 1.2 years. Six patients were positive for rheumatoid factor and 3 patients were positive for ACPA. 2 patients were positive for anti-SS-A antibody and 3 patients were positive for anti-SS-B antibody. The total times of MSUS were 19 times, and 560 joints/128 entheseal sites were scanned. In 6 patients, MSUS revealed abnormal findings to 42 of 560 joints (35/286 joints of ACPA positive patients vs. 7/274 joints of ACPA negative patients, P<0.001). Power Doppler (PD) signal was found at 28 joints in 3 patients with ACPA, while no PD signal was detected in 5 patients without ACPA. One patient developed arthritis with PD signal in the course of illness. Tenosynovitis was detected at 15 joints in 5 patients. Active enthesitis with PD signal and bursitis were found at Achilles tendons in 2 patients with ACPA. By MSUS, subclinical arthritis was found in some joints (to 23 in 286 joints of ACPA positive patients vs. to 4 in 274 of ACPA negative patients, P=<0.001). On the other hand, there found no abnormality in 67 swelling and/or painful joints by MSUS (31/286 joints of ACPA positive patients vs. 36/274 joints of ACPA negative patients, P=0.40). Bone erosion was not detected in our cases. From this study, we also did not found calcifications nor enthesophytes even on joints with PD signal positive arthritis or enthesitis. This result is comparable to past report that arthritis with SjS are usually non-erosive. However, it is capable that this result is just because of being in early phase or we started treatment promptly, we have to observe their course deliberately.

## Conclusion

We suggest that juvenile primary SjS patients with ACPA have risks of arthritis with PD signal, and sometimes combined with enthesitis. Since there is no prediction whether to complicate joint destruction or not, we should take account of structural lesions by regular imaging.

## Disclosure of interest

None declared.

